# Exosomes in Central Nervous System Diseases: A Comprehensive Review of Emerging Research and Clinical Frontiers

**DOI:** 10.3390/biom14121519

**Published:** 2024-11-27

**Authors:** Jingrun Li, Jiahao Song, Lina Jia, Mengqi Wang, Xunming Ji, Ran Meng, Da Zhou

**Affiliations:** 1 Department of Neurology, Xuanwu Hospital, Capital Medical University, Beijing 100053, China; 2 Advanced Center of Stroke, Beijing Institute for Brain Disorders, Beijing 100053, China; 3 National Center for Neurological Disorders, Xuanwu Hospital, Capital Medical University, Beijing 100053, China

**Keywords:** exosomes, central nervous system diseases, blood-brain barrier, neuroprotection, therapeutic applications

## Abstract

Exosomes, nano-sized lipid bilayer vesicles, have garnered significant attention as mediators of cell communication, particularly within the central nervous system (CNS). Their unique properties, including high stability, low immunogenicity, and the ability to traverse the blood-brain barrier (BBB), position them as promising tools for understanding and addressing CNS diseases. This comprehensive review delves into the biogenesis, properties, composition, functions, and isolation of exosomes, with a particular focus on their roles in cerebrovascular diseases, neurodegenerative disorders, and CNS tumors. Exosomes are involved in key pathophysiological processes in the CNS, including angiogenesis, inflammation, apoptosis, and cellular microenvironment modification. They demonstrate promise in mitigating ischemic injury, regulating inflammatory responses, and providing neuroprotection across various CNS conditions. Furthermore, exosomes carry distinct biomolecules, offering a novel method for the early diagnosis and monitoring of CNS diseases. Despite their potential, challenges such as complex extraction processes, the heterogeneity of exosomal contents, and targeted delivery limitations hinder their clinical application. Nevertheless, exosomes hold significant promise for advancing our understanding of CNS diseases and developing novel therapeutic strategies. This manuscript significantly contributes to the field by highlighting exosomes’ potential in advancing our understanding of CNS diseases, underscoring their unique value in developing novel therapeutic strategies and mediating cellular communication.

## 1. Introduction

The blood-brain barrier (BBB) has long posed a significant challenge for central nervous system (CNS) research, particularly regarding the delivery of therapeutic agents. Recent advancements in science and technology have led to the discovery of numerous nanomaterials, spurring innovations in both therapeutic and diagnostic strategies. Among these, exosomes, a subclass of nanomaterials, have garnered considerable attention for their potential in theranostic applications due to their multifaceted cellular effects [1].

Exosomes, which originate from endocytosis, are secreted by nearly all cell types and are present in various extracellular fluids [2,3,4,5,6]. Initially identified in sheep reticulocytes by Johnstone et al. over three decades ago and regarded merely as cellular debris, the perception of exosomes has dramatically evolved [7]. It is now recognized that exosomes facilitate intercellular communication, influencing a wide array of biological processes, including angiogenesis, inflammation, apoptosis, and the modulation of the cellular microenvironment [5,8,9]. Notably, exosomes can traverse the BBB under certain conditions, underscoring their potential to affect CNS pathophysiological processes and serve as viable drug carriers for treating CNS disorders [10,11]. This review provides a comprehensive analysis of the biological characteristics of exosomes and summarizes the current research dedicated to their application, with a particular focus on the relationship between exosomes and CNS disorders.

## 2. Biological Characteristics of Exosomes

### 2.1. Biogenesis

**Figure 1:**
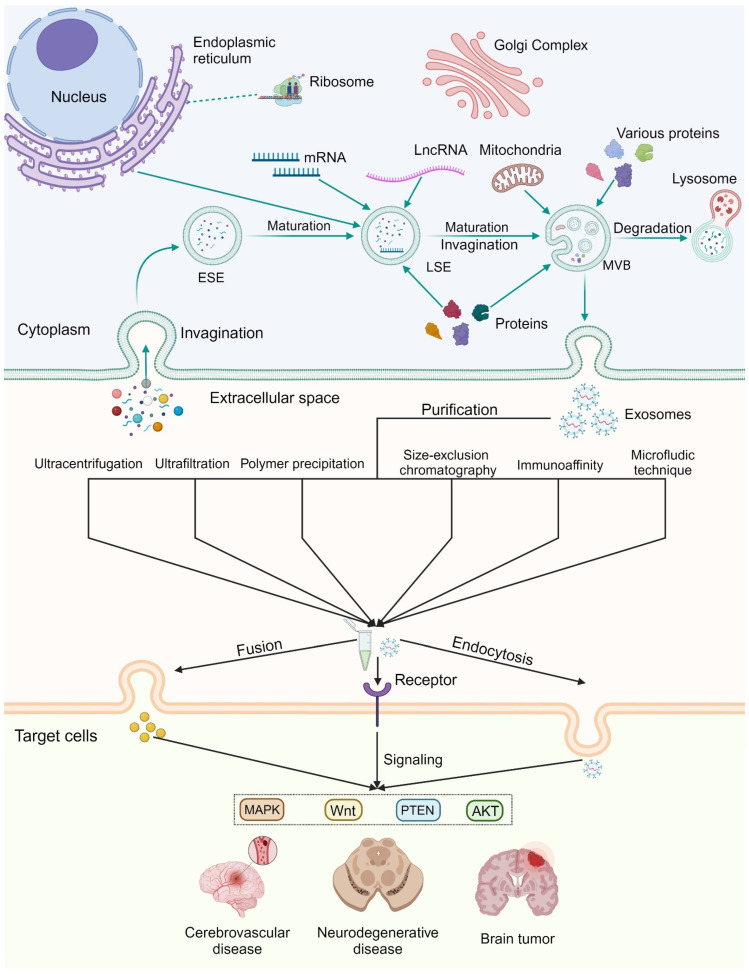
Exosomal biogenesis and pathways to biological roles. Exosomes originate from endocytosis. During this process, invagination of the plasma membrane transforms ESEs into LSEs through multiple folding and chemical reactions. Coordinated with Rab, GTPase, and SNARE proteins, MVBs fuse with the plasma membrane to release exosomes. Exosomes are characterized by their heterogeneity, necessitating efficient isolation and purification techniques. They fulfill biological roles through fusion, endocytosis, and interaction with receptors, thereby activating various signaling pathways involved in central nervous system diseases. Abbreviations: ESE, early sorting endosomes; LSE, late sorting endosomes; MVB, multivesicular bodies.

Exosomes are generated from the endocytic pathway [12]. This process begins with the invagination of the plasma membrane, resulting in the formation of early sorting endosomes (ESEs) [5,13,14]. These ESEs mature by incorporating various extracellular components and membrane proteins, and transform into late sorting endosomes (LSEs). The acidification of LSEs, primarily regulated by Rab5 and Vps34/P150, is crucial for their maturation [15,16]. Afterwards, LSEs evolve into multivesicular bodies (MVBs), which have two possible fates: degradation via lysosomes or fusion with the plasma membrane to release exosomes into the extracellular space [17,18]. Although the exact mechanism underlying exosome formation is complex and not fully established, prevailing theories highlight the roles of the endosomal sorting complex required for transport (ESCRT)-dependent and ESCRT-independent pathways. Additionally, four-transmembrane domain proteins and lipid rafts are believed to participate in exosome formation (Figure 1) [19].

### 2.2. Properties

Exosomes, a class of extracellular vesicles ranging from 40 to 150 nm in diameter, are identifiable in a myriad of body fluids, including blood, lymphatic fluid, saliva, urine, semen, and breast milk [20]. Characterized predominantly by the lipid-to-protein ratio, their density falls between 1.13 and 1.19 g/mL, as determined through density gradient centrifugation [21,22]. Under transmission electron microscopy, exosomes typically present as cup-shaped entities but appear as round vesicles under cryo-electron microscopy [23]. The morphology of exosomes can vary even when derived from the same cell type, highlighting their heterogeneity and the importance of developing refined isolation techniques based on their physical properties [24].

### 2.3. Composition

**Figure 2:**
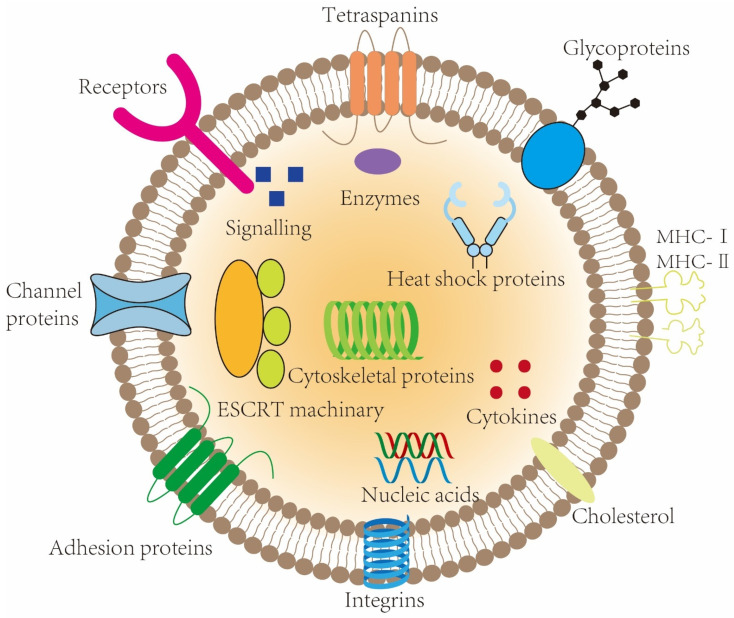
Exosomal structure. Exosomes are stable bilayer membrane vesicles. The contents of different exosomes vary considerably. Generally, exosomes contain three main types of substances: proteins, lipids, and nucleic acids. Proteins are distributed on and within the membranes and play roles in exosomal formation and signaling. The nucleic acids in exosomes are mainly RNA, although some DNA is also present. The lipids are primarily located in the membrane, enhancing the stability of the exosomal membrane and assisting in signaling. Abbreviations: ESCRT, endosomal sorting complex required for transport; MHC, major histocompatibility complex.

The composition of exosomes reflects the dynamic state of their originating cells, embodying a complex array of proteins, lipids, and nucleic acids (Figure 2) [25,26]. This compositional variability endows exosomes with a broad spectrum of biological functionalities [8]. Proteins within exosomes, whether ubiquitous or cell-specific, are derived from processes such as endocytosis, membrane fusion, and cytoplasmic activities [27,28,29]. Ubiquitous proteins, including cytoskeletal proteins, tetraspanin, and GTPases, are essential for the structural integrity and functional efficacy of virtually all exosomes [15,30,31]. Conversely, cell-specific proteins, dictated by the exosome’s cellular origin, confer unique functional attributes tailored to specific physiological or pathological contexts [15,32,33].

Additionally, exosomes are rich in various forms of RNA, including messenger RNAs, long non-coding RNAs, and transfer RNAs. These nucleic acids can be transcribed and translated or even directly integrated into the genome of recipient cells, thus profoundly influencing their phenotypic characteristics [34,35]. The strategic inclusion of RNAs within exosome cargoes, as highlighted by Fabbiano et al., mirrors the state of the originating cell, which underscores the utility of exosomal RNAs in diagnostic applications [36,37]. Moreover, the lipid makeup of exosomes—distinct from that of their parental cells and enriched in cholesterol, sphingolipids, phosphatidylserine, and ceramides [35]—fortifies the stability of the exosomal membrane, further exemplifying the intricate design and functional capacity of these vesicles [38,39].

### 2.4. Functions and Advantages

Exosomes serve as versatile nanoplatforms, orchestrating the delivery of biomolecular cargoes, thereby regulating intercellular communication and modulating the behavior of recipient cells [13,40]. Their functional diversity is attributed to their origin, size, and varied constituents [41]. Exosomes can either promote or inhibit disease progression, rendering them promising for both diagnostic and therapeutic applications [42,43]. The presence of exosomes in various body fluids offers a novel approach to the early diagnosis and monitoring of a range of disorders. Furthermore, the innate biocompatibility of exosomes, combined with their ability to traverse biological barriers such as the BBB, positions them as invaluable therapeutic agents. They provide several advantages, including extending drug half-lives, minimizing systemic toxicity, and enhancing targeting efficacy [44].

### 2.5. Isolation

As the potential of exosomes continues to be explored, related separation technologies are rapidly advancing, effectively supporting their clinical use. Based on their physicochemical properties and composition, multiple purification technologies have been developed, including ultracentrifugation, ultrafiltration, size-exclusion chromatography, polymer precipitation, immunoaffinity capture, and integrated microfluidic technique (Figure 1) [45]. Ultracentrifugation is considered the gold standard method due to its ability to generate high centrifugal forces and eliminate the need for exosome labeling [46]. Recent advancements have led to superior separation methods through improvements or combinations of existing technologies [47]. For example, Chen et al. introduced double-coupled harmonic oscillations into ultrafiltration, improving processing speed, yield, and purity [48]. Despite significant advances in exosome isolation techniques, researchers still encounter challenges such as complex extraction procedures and low yields [49].

## 3. Exosomal Effects in Cerebrovascular Diseases

### 3.1. Ischemic Cerebrovascular Disease

Ischemic cerebrovascular disease (CVD) is a leading cause of mortality and disability worldwide, representing a significant health concern [50,51]. Neuronal death in the ischemic hemispheric zone occurs swiftly after the onset of an ischemic stroke, making the interaction between exosome and neuronal death a critical area of study in cerebral ischemia [52,53]. Exosomes exhibit notable effects on multiple cell death pathways, including apoptosis, pyroptosis, and ferroptosis [54]. Neural progenitor cell-derived exosomes reduce neural apoptosis through the NADPH oxidase 2 (Nox2)/reactive oxygen species (ROS) and brain-derived neurotrophic factor (BDNF)/ tyrosine kinase receptor B (TrkB) pathways. This effect is attributed to the overexpression of miR-126 and miR-210, which diminishes ROS production and promotes dendritic spine density [55]. Further studies indicate that exosomal opa interacting protein 5-antisense RNA 1 (OIP5-AS1) inhibits neuronal pyroptosis by relying on thioredoxin-interacting protein (TXNIP) ubiquitination and degradation [56]. Recently, ferroptosis has garnered attention due to its roles in the prognosis of ischemic stroke [54]. Exosomes from adipose-derived mesenchymal stem cells carrying miR-760-3p have proven to be effective anti-ferroptosis strategies. These exosomes can inhibit the expression of ChaC glutathione specific gamma-glutamylcyclotransferase 1 (CHAC1), a key gene in the progression of ferroptosis [57].

In the context of the inflammatory response following ischemia, exosomes emerge as vital mediators, particularly in modulating inflammasome activity. After the onset of ischemic stroke, impaired brain tissues generate a series of damage-associated molecular patterns (DAMPs), which activate immune cells [58,59]. Exosomes are crucial carriers of DAMPs, [60] and inhibiting exosomes may prevent the diffusion of DAMPs [61]. Exosomes not only assist in DAMP diffusion but also mediate their production [62]. Exosomal cargoes such as programmed death-ligand 1 (PD-L1) can retard necrosis, alleviating the burst of DAMPs at its source [63]. Conversely, exosomal modification to DAMPs facilitates their secretion and release [64]. Immune cells, especially microglia, are involved in the initiation and progression of inflammation [65]. Exosomes can shift the phenotype of activated microglia to either the “pro-inflammation” M1 type or the “anti-inflammation” M2 type [66,67]. For instance, exosomes promote the conversion of microglia to an M1 phenotype through the Roquin-interferon regulatory factor 4 (IRF4) axis governed by exosomal miR-3613-3p [68,69]. Exosomes can activate the nuclear factor-kappa B (NF-κB) signaling pathway by acting on toll-like receptors (TLRs), thus promoting the polarization of macrophages to the M1 phenotype [70,71]. Furthermore, evidence suggests that exosomes can regulate the function of macrophages, as exemplified by their ability to counteract PAMP-induced macrophage activation through the prostaglandin E2 (PGE2)/cAMP response element-binding protein (CREB) pathway [72].

**Figure 3:**
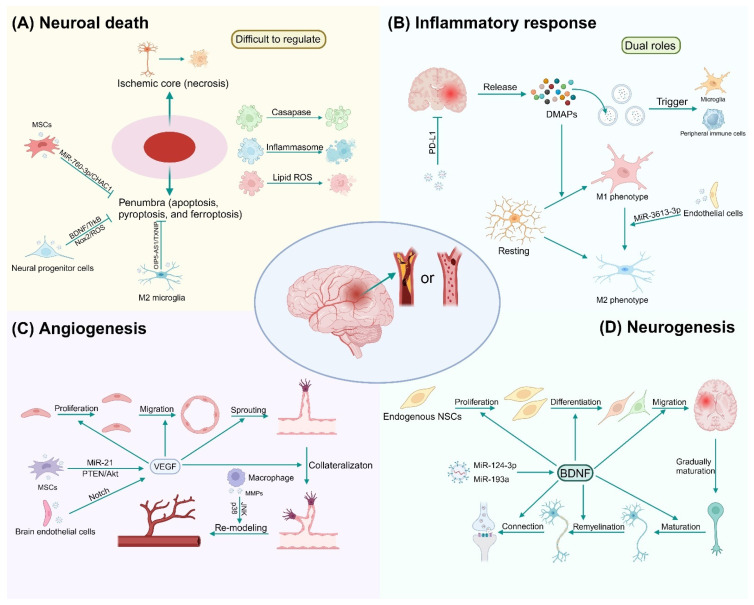
Exosomal effects in cerebrovascular diseases. Exosomes can influence the progression of cerebrovascular diseases in various ways. They can accelerate the deterioration or delay the progression of these diseases. Exosomes exert effects mainly through mechanisms involving neuronal death, inflammation, angiogenesis, and neurogenesis. Abbreviations: MSCs, mesenchymal stem cells; ROS, reactive oxygen species; BDNF, brain-derived neurotrophic factor; TrkB, tropomyosin receptor kinase B; Nox2, NADPH oxidase 2; PD-L1, programmed cell death 1 ligand 1; DAMPs, damage-associated molecular patterns; VEGF, vascular endothelial growth factor; MMP, matrix metalloproteinase; JNK, c-Jun N-terminal kinase; NSCs, neural stem cells.

Recent animal studies have progressively illuminated the crucial role exosomes play in the pathogenesis of ischemic CVD (Figure 3). These vesicles have demonstrated a profound capacity to mitigate brain damage at ischemic sites by promoting angiogenesis—a vital mechanism for recovery after ischemic injury [73]. For instance, macrophage-derived exosomes have been identified to upregulate the secretion of matrix metalloproteinases (MMPs), essential for vascular remodeling and repair via the c-JunN-terminal kinase (JNK) and p38 pathways [74,75]. Exosomes contain a variety of bioactive molecules imperative for endothelial cell (EC) proliferation and migration, elements fundamental to angiogenesis [76]. Specifically, exosomal miRNAs such as miR-21, augment angiogenesis by increasing vascular endothelial growth factor (VEGF) levels reliant on a phosphatase and tensin homolog deleted on chromosome ten (PTEN)/protein kinase B (Akt)-dependent mechanism [77,78,79]. Additionally, exosomes from brain ECs, through the activation of the Notch signaling pathway, collaborate with pericytes to bolster angiogenesis and maintain BBB integrity [80]. This interaction also protects pericytes from pyroptosis, facilitating the repair of ischemic damage [81].

Neurogenesis can occur after various central nerve injuries, being especially important for the improvement of neural function after stroke [82]. Emerging studies manifest that exosomes partly mediate the rehabilitation of infarct focus. Exosomes first promote angiogenesis and lay a solid material foundation for subsequent neurogenesis. Later, exosomes promote neural stem cell recruitment and neuronal differentiation. Processes such as axonal regeneration, remyelination, and synapse formation are subsequently launched, eventually resulting in neurogenesis and even brain structural remodeling [83,84,85]. During neurogenesis, exosomes carry BDNF to promote neuroplasticity [86]. Exosomes regulate neurogenesis mainly by miRNAs. MiR-124-3p is identified with the highest enrichment in neuron-derived exosomes involved in neurogenesis [87]. Further research shows that miR-124-3p may act on the phosphoinositide 3-kinase (PI3K)/AKT/NF-κB signaling pathway. Increased levels of exosomal miR-193a are detected after neuronal differentiation [88], further implying the relationship between exosomal miRNAs and neurogenesis.

### 3.2. Hemorrhagic Cerebrovascular Disease

Non-traumatic intracerebral hemorrhage (ICH) is considered the most lethal type of stroke, with a mortality of exceeding 50% within one year [89]. Despite available medical or surgical interventions, improving the prognosis of ICH remains a formidable challenge [90,91]. Recent research suggests exosomes as potential innovative therapies for ICH. For instance, using a mouse model of ICH, Li et al. documented an increase in exosome levels in the brain, finding that suppression of their release exacerbated neurological damage [92]. The therapeutic effects of exosomes are largely dependent upon their cargo. Lai et al. discovered that delivering exosomal miR-193b-3p to mice with subarachnoid hemorrhage (SAH) ameliorated subsequent neuroinflammatory responses and neurobehavioral impairments, presumably by downregulating histone deacetylase 3 (HDAC3) expression and upregulating NF-κB p65 acetylation [93]. Another study demonstrated that exosomal miR-124 could restrict microglial activation and neuroinflammation, thereby alleviating secondary brain injury in a SAH rat model [94]. BDNF is known to exert critical functions in mediating neuronal survival in various diseases, including ICH. Further investigation has shown that BDNF transferred by mesenchymal stem cell (MSC)-derived exosomes can attenuate apoptotic neuronal death and enhance neurogenesis following intraventricular hemorrhage in vivo [95]. Nevertheless, as these exploratory studies are mainly conducted on small animals, the therapeutic value of exosomes in ICH remains uncertain.

### 3.3. Venous Thromboembolism

Cerebral venous thrombosis (CVT), a rare but significant type of stroke, disproportionately affects young individuals with a female predominance [96,97]. The heterogeneity in clinical manifestations, myriad a risk of factors, lack of standardized treatment protocols, and unpredictable patient outcomes complicate the diagnosis and management of CVT [98].

Exosomes have been implicated in the pathogenesis of venous thrombosis, potentially regulating thrombus formation by modulating vascular EC activation, primarily through mechanisms driven by oxidative stress and ROS [99]. Crewe et al. observed that adipocyte-derived exosomes carrying oxidatively damaged mitochondrial particles could induce ROS surges in recipient cells [100]. Conversely, Zhang et al. demonstrated that exosomal miR-522 could suppress ROS production by inhibiting Arachidonate-15-Lipoxygenase (ALOX15), highlighting the dual role of exosomes in oxidative stress modulation [101]. Moreover, exosomes influence EC activity by impacting key regulators such as P53, caspase-3, and Plasminogen activator inhibitor-1. The intimate association between platelets and exosomes, and their regulation of venous thromboembolism (VTE) through exosome secretion has been documented [102]. In a previous study, the colchicine-mediated inhibition of platelet exosome release was found to significantly lower prothrombin levels [103]. Exosomes also orchestrate the systemic inflammatory response integral to VTE pathogenesis by recruiting inflammatory cells and releasing inflammatory factors.

Although D-dimer levels are traditionally used to predict thrombotic events, their specificity is limited. Exosomes bearing a multitude of biological markers may offer enhanced diagnostic precision. Han et al. pinpointed exosomal peroxidase as a novel marker for VTE diagnosis [104]. The differential expression of circulating miRNAs, such as miR-522-3p and miR-134 between VTE patients and healthy controls further suggests a potential diagnostic utility [105].

In the therapeutic domain, exosomes hold promise for VTE management, with evidence revealing that exosomal components could deter VTE progression or even reverse it. Notably, exosomal miR-233 has been shown to counteract atherosclerosis and vascular endothelial inflammation, with similar therapeutic potentials observed for miR-342-3p and miR-339 [106,107]. Additionally, the adjunctive use of exosomes in existing therapies, exemplified by the deployment of exosome-eluting stents, underscores their potential to reduce endothelial hyperplasia, mitigate inflammation, and lower the risk of thrombotic recurrence [108].

While research on the relationship between exosomes and CVT is limited, valuable insights can be drawn from studies on other venous thromboses. Given the shared pathogenesis between CVT and other forms of venous thrombosis, there is a high likelihood that exosomes could be applicable to CVT. With their ability to cross the BBB, exosomes may herald a new era in CVT research. The main effects of exosomes in the progression of cerebrovascular diseases are detailed in Table 1.

## 4. Brain Tumor

Brain tumors, although relatively rare, are notably lethal, especially among children and adolescents, where they are responsible for a substantial proportion of cancer-related deaths [109]. Exosomes may play a key role in the development and potential treatment of brain tumors.

### 4.1. Exosomes in Brain Tumor Development

The tumor microenvironment (TME) of brain tumors, comprising the extracellular matrix, blood vessels, stromal cells (e.g., immune cells, fibroblasts, ECs, mesenchymal stem cells), and a milieu of secreted factors (e.g., cytokines, growth factors), profoundly influences tumor progression [110,111]. Recent research has spotlighted the ability of exosomes to remodel the TME, thereby facilitating tumor development through mechanisms including angiogenesis, immune evasion, and the provision of stimulatory signals (Figure 3).

Angiogenesis, a process that supplies oxygen and nutrients to tumor cells and contributes to tumor metastasis and recurrence [112], is recognized as a hallmark of cancer [111]. Exosomes laden with angiogenic factors like VEGF, fibroblast growth factor (FGF), interleukin-6 (IL-6), and interleukin-8 (IL-8) [15] promote the migration and proliferation of vascular endothelial cells (VECs), thus bolstering angiogenesis. Moreover, exosomal miRNAs, including miR-135b, miR-210, and miR-21, are implicated in modulating the biological behavior of these ECs [113,114]. Beyond affecting VECs, exosomes also act on other components of the TME, such as macrophages, MSCs, and fibroblasts, to release pro-angiogenic signals [115,116,117].

Tumor cell-derived exosomes carrying oncogenic factors seed the TME with conditions conducive to tumor growth. The secretion of exosomes is directly proportional to tumor aggressiveness, highlighting their role in cancer biology [118,119]. For example, glioblastoma-derived exosomes containing the Notch1 protein can induce carcinogenesis in normal glial cells through Notch1 signaling activation [119]. The inflammatory milieu within the TME [120], augmented by the exosomal secretion of transforming growth factor beta (TGF-β), interleukin-4 (IL-4), and interleukin-10 (IL-10), modulates the local immune response to favor tumor progression [121].

The interaction between tumor cells and the immune system is intricately mediated by exosomes within the TME [122], leading to an immunosuppressive environment that facilitates tumor development [123]. Exosomal PD-L1, for instance, binds to programmed death-1 (PD-1) on CD8+ T lymphocytes, attenuating their activity and proliferation [124]. Exosomes also downregulate the expression of co-stimulatory molecules (e.g., CD27), further inhibiting T cell activation [125]. In response to exosomal signals, macrophages may undergo polarization to the M2 phenotype, which supports tumor growth and metastasis [126]. Additional studies have demonstrated that tumor cell-derived exosomes extend the survival of M2 macrophages within the TME [127] and similarly modulate other immune cells (e.g., NK cells, dendritic cells, myeloid-derived suppressor cells), resulting in overall immunosuppression [128].

Besides remodeling the TME, exosomes directly interact with tumor cells to facilitate the development of drug resistance [118]. Ding et al. reported that exosomal circ_0072083 promotes temozolomide resistance in glioma by regulating miR-1252-5p-mediated degradation and demethylation [129]. Cells may develop resistance to therapeutic agents by absorbing exosomes from cells already resistant to those treatments [130,131]. This discovery points to the complex role exosomes play in mediating cellular communication and drug resistance, emphasizing their significance in understanding cancer’s adaptive mechanisms and in devising innovative therapeutic strategies to counteract tumor resilience.

### 4.2. Exosomes in the Diagnosis and Treatment of Brain Tumors

**Figure 4:**
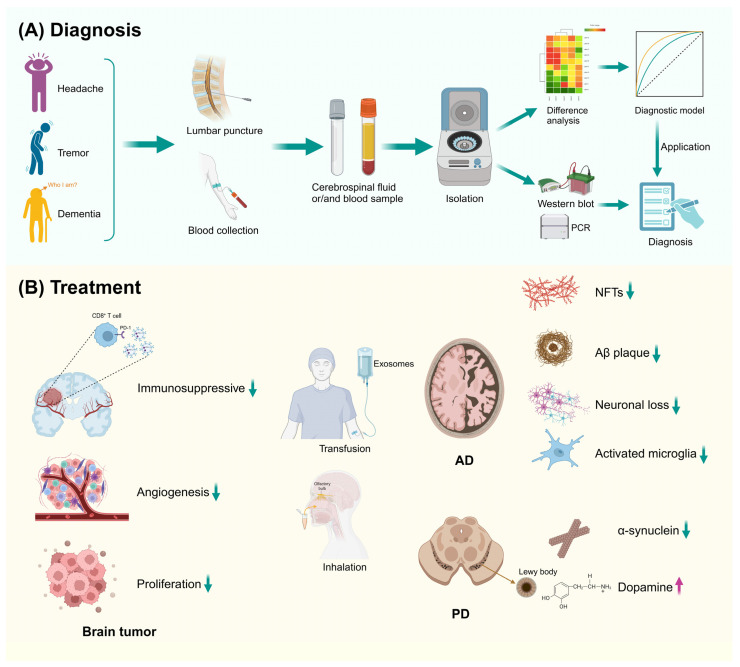
Exosomal application in the diagnosis and treatment of brain tumors, AD, and PD. (**A**) Exosomes are present in various extracellular fluids, making them accessible from blood and cerebrospinal fluid samples. After purification, their physical and chemical properties are analyzed to diagnose diseases. (**B**) Exosomes, secreted by almost all cells, can cross the blood-brain barrier. Consequently, they are widely used in the treatment of brain tumors, AD, and PD. Abbreviations: AD, Alzheimer’s disease; PD, Parkinson’s disease; Aβ, amyloid β-protein.

Exosomes, ubiquitous in body fluids, facilitate liquid biopsies by carrying distinct biomolecules from healthy individuals to patients with brain tumors (Figure 4) [132]. Their cargo, particularly miRNAs stabilized by exosomal membranes, can act as crucial biomarkers for diagnosing brain tumors [4]. Lan et al. observed that glioma patients exhibit significantly elevated serum levels of exosomal miR-301a compared to controls [133]. Furthermore, the differential presence of exosomal miR-766-5p elevates its utility in distinguishing glioma from conditions such as intracranial lymphoma [134]. The correlation between exosomal miR-210 levels and brain tumor malignancy further underscores the prognostic value of exosomal miRNAs [135].

The effective delivery of anticancer drugs is often hindered by obstacles such as poor solubility, limited bioavailability, and the inability to penetrate the BBB [40]. Exosomes emerge as promising vectors capable of overcoming these barriers, facilitating drug delivery directly to brain tumors. Their inherent stability, subtle negative surface charge, and specific membrane components, such as glycosaminoglycans derived from glioma exosomes, significantly enhance their targeting efficiency [136]. The acidic microenvironment of tumors and the enhanced permeability and retention effect in solid tumors further improve exosome-mediated delivery, optimizing drug targeting to malignant cells [137,138].

Exosomes also mediate intercellular communication that can accelerate tumor growth and metastasis. Inhibiting tumor cell secretion of exosomes may decelerate tumor progression, making the identification of efficient and selective exosome inhibitors a vital research avenue. The association of Rab proteins with exosome secretion positions compounds like tipifarnib as potential inhibitors, which reduce the release of exosomes and thereby their contribution to tumor development [139]. Additionally, targeting sphingomyelinase with specific inhibitors such as Manumycin A offers another therapeutic strategy to suppress tumor-derived exosome secretion [140]. The main effects of exosomes in the progression of brain tumors are detailed in Table 2.

## 5. Neurodegenerative Diseases

Neurodegenerative diseases, including Alzheimer’s disease (AD), Parkinson’s disease (PD), multiple sclerosis (MS), and Huntington’s disease (HD), are characterized by progressive neuronal loss and functional decline, leading to cognitive and behavioral impairments [141]. According to the World Health Organization, these diseases are projected to become the second leading cause of death globally [142]. Exosomes are increasingly recognized for their critical roles in the pathogenesis, diagnosis, and treatment of neurodegenerative diseases, positioning them at the forefront of current research in this field.

### 5.1. Alzheimer’s Disease

The pathology of AD is marked by the accumulation of amyloid-beta (Aβ) and neurofibrillary tangles (NFT), formed by hyperphosphorylated Tau protein [143]. Exosomes play a dual role in AD, contributing to pathology while also holding promise for diagnostic and therapeutic applications (Figure 4). They are implicated in the cleavage of amyloid precursor proteins (APPs) through β-secretase, a crucial step in Aβ production [144]. Moreover, exosomal miRNAs, notably miR-101, can modulate APP expression and β-secretase activity, thereby affecting Aβ aggregation [145,146]. The presence of exosomal proteins like Alix in amyloid plaques of AD patients further signifies the significance of exosomes in Aβ aggregation [146]. Additionally, the interaction between exosomes and Aβ, facilitated by ceramides within exosomes, promotes Aβ aggregation [147].

Tau pathology, another hallmark of AD, involves the transformation of normal tau proteins into hyperphosphorylated variants, culminating in NFT formation [148]. Exosomal miRNAs, such as miRNA-200c, have been shown to contribute to increased tau phosphorylation [149]. Research indicates that phosphorylated Tau proteins are enriched in exosomes secreted by the brain tissue of AD patients, suggesting their involvement in the effective transmission of Tau proteins [150,151].

Lysosomal dysfunction, linked to neuronal loss in AD [152], promotes exosomal secretion to eliminate toxic proteins. This process exacerbates the accumulation of extracellular toxic proteins, predominantly Aβ metabolites [153]. Upon internalization by other cells, these proteins can impair cellular mitochondria and ion channels, leading to cell death [154,155]. Furthermore, neuron-derived exosomes in AD patients exhibit significantly reduced levels of cell survival factors, such as low-density lipoprotein receptor-related protein 6 (LRP6) and heat shock factor-1 (HSP-1), compared to controls [156]. Exosomes are also related to intracranial inflammation, evidenced by elevated levels of complement components and pro-inflammatory factors in cerebrospinal fluid (CSF)-derived exosomes from AD patients, signifying their contribution to disease progression [157].

Exosomes derived from neurons and astrocytes offer diagnostic markers for AD, [158] enabling early detection and monitoring before symptom onset through the quantification of Aβ and tau protein levels [159]. These proteins in neuron-derived exosomes correlate with their levels in CSF, which indicate that the diagnostic accuracy is comparable to that of CSF analysis [160]. Emerging research has identified exosomal proteins (e.g., cathepsin D and synaptosome-associated proteins) [161,162,163], and miRNAs (e.g., miR-30b-5p, miR-22-3p, miR-378a-3p) as markers for early AD diagnosis [164].

Therapeutically, exosomes can mediate the degradation of Aβ through various pathways, including direct breakdown by enzymes such as enkephalinase and insulinase [165,166], or indirectly by promoting Aβ clearance via lymphatic drainage and BBB transport [144]. Sphingolipids in exosomes can induce conformational changes in Aβ deposits, facilitating their uptake and breakdown by glial cells [167].

AD is often accompanied by pathological features such as oxidative stress and synaptic dysfunction, which can cause severe neuronal loss [168]. Exosomes have a neuroprotective role, potentially improving the prognosis of AD patients by suppressing inflammation through the regulation of glial cell function [169]. MSC-derived exosomes have been shown to protect hippocampal neurons by inhibiting oxidative stress induced by Aβ, possibly involving exosomal peroxidase [170]. Another study demonstrated that exosomes secreted by neural stem cells could safeguard synapses against Aβ binding [171]. Moreover, Wei et al. discovered that MSC-derived exosomal miR-223 offered protection to neuronal cells from apoptosis through the PTEN-PI3K/Akt pathway [172]. The main effects of exosomes in the progression of AD are shown in Table 3.

### 5.2. Parkinson’s Disease

PD is delineated by the aggregation of α-synuclein (α-syn), which leads to the formation of Lewy bodies and a pronounced loss of dopaminergic neurons in the substantia nigra pars compacta [173]. Exosomes have been identified as key players in the transmission of α-syn, [174] enabling its transport across long distances and inducing substantial neurotoxic effects (Figure 4) [175]. The presence of α-syn in exosomes enhances cellular uptake and drives neuroinflammation, ultimately resulting in neuronal death [176]. Moreover, exosomal miRNAs, such as miR-21 and miR-146a, are known to trigger inflammatory responses through the NF-κB/NLR family pyrin domain containing 3 (NLRP3) pathway, which further aggravates PD progression [177].

The transportation of miRNAs and aberrant proteins by exosomes sheds light on the mechanisms underlying PD progression and unveils potential therapeutic targets [178]. Shi et al. reported a correlation between exosomal α-syn levels and the severity of PD, with these levels being substantially higher than those in serum [179]. Comparative protein analysis of plasma exosomes from PD patients revealed significantly reduced levels of clusterin, complement C1r subcomponents, and apolipoprotein A1 compared to healthy individuals [180]. Additionally, exosomal miRNA profiling indicated a marked elevation in miR-331-5p levels, alongside a decrease in miR-505, suggesting their utility as biomarkers for PD [181].

Current treatments for PD primarily address symptoms without offering a cure. The capability of exosomes to ferry active substances across the BBB presents a promising therapeutic avenue. Qu et al. illustrated that exosomes could effectively deliver dopamine to the brain, leveraging interactions between transferrin and its receptors [182]. Exosomes are also capable of transporting genetic material that can regulate gene expression [183]. Kojima et al. observed significant anti-inflammatory effects in rat models of PD through the delivery of hydrogen peroxide via exosomes [184]. Furthermore, Izco et al. reported the successful inhibition of α-syn aggregation in PD rat models by utilizing exosomes to convey short hairpin RNA (shRNA) [185].

### 5.3. Other Neurodegenerative Diseases

Neurodegenerative diseases encompass a spectrum of conditions marked by progressive CNS damage [186]. Despite their varied etiologies, these disorders share common pathological hallmarks, including abnormal protein aggregation, neuroinflammation, oxidative stress, and neuronal loss [187]. Exosomes, pivotal in cell-to-cell communication, play significant roles across various physiological and pathological contexts, rendering them indispensable to neurodegenerative disease research [22]. For instance, exosomes have been identified as carriers of HD proteins, establishing them as a potential diagnostic biomarker [188]. Moreover, research in MS models has revealed that exosomal let-7i miRNA can influence the disease’s pathogenesis by targeting the insulin-like growth factor 1 receptor (IGF1R)/TGF-β type 1 receptor (TGFBR1) signaling pathway, which underscores its therapeutic relevance [189]. The main effects of exosomes in the progression of PD, HD, and MS are detailed in Table 4.

## 6. The Roles of Exosomes in Other Central Nervous System Diseases

Exosomes have emerged as key players in CNS diseases, attributed largely to their ability to traverse the BBB. This unique characteristic positions them as a vital tool in the study and treatment of CNS injuries, particularly by modulating processes such as apoptosis, inflammation, and angiogenesis [190]. Guo et al. demonstrated the therapeutic potential of exosomes by administering them with phosphatase and siRNA to rats suffering from spinal cord injuries. These exosomes were found to substantially foster axonal regeneration and angiogenesis while concurrently reducing the proliferation of microglia and astrocytes [191]. Likewise, Manek et al. observed that patients with severe cranial injuries exhibited increased levels of exosomes associated with cell death and neurodegeneration following brain injury. These findings suggest that targeting the release of such exosomes could be a promising strategy to improve the prognosis for individuals with CNS injuries [192].

## 7. Clinical Challenges and Perspectives

The transition of exosome research from bench to bedside in the context of CNS diseases is at a pivotal juncture, fraught with challenges yet brimming with potential. The multidimensional utility of exosomes as diagnostic tools, therapeutic vehicles, and biomarkers offers a promising frontier in neurology. However, several significant barriers to clinical translation persist, necessitating focused efforts to overcome them.

### 7.1. Complex Extraction and Scalability Issues

A primary concern in the clinical application of exosomes is the complexity of their extraction and purification processes. Traditional methods such as ultracentrifugation, while widely employed, often co-isolate non-exosomal extracellular vesicles, introducing variability and potentially confounding experimental outcomes. Advanced techniques, including size-exclusion chromatography and immunoaffinity capture, although offering enhanced specificity, are labor-intensive and challenging in terms of scalability. These methodological disparities underscore the pressing need for the development and standardization of protocols to ensure the reproducibility, purity, and scalability of exosome preparations for clinical use.

### 7.2. Therapeutic Targeting and Delivery

The inherent ability of exosomes to traverse biological barriers, including the BBB, heralds their potential as vehicles for CNS-targeted therapies. However, the engineering of exosomes for enhanced targeting specificity to diseased cells or tissues remains a challenge. Innovations in exosome surface modification techniques, such as the incorporation of targeting ligands or antibodies, offer a path forward. These modifications aim to improve the homing efficiency of therapeutic exosomes, ensuring their accumulation at the site of pathology without off-target effects.

### 7.3. Regulatory and Safety Considerations

The regulatory landscape for exosome-based therapeutics remains nascent, with clear guidelines for production, characterization, and quality control yet to be fully established. Additionally, while exosomes are generally considered to have low immunogenicity, the potential for immune responses, particularly when using allogeneic exosomes, cannot be entirely discounted. Rigorous long-term safety and immunogenicity studies are essential to elucidate and mitigate potential risks associated with exosome-based therapies.

### 7.4. Clinical Trials

Recent advancements in exosome research have catalyzed the initiation of several promising clinical trials, as detailed in Table 5. For instance, an ongoing clinical trial (NCT06082713) is at the forefront, evaluating the safety and preliminary efficacy of intravenously administered exosomes derived from human induced pluripotent stem cells (GD-iExo-003) in acute ischemic stroke. This endeavor represents a significant step toward harnessing the therapeutic potential of exosomes in mediating recovery after stroke. Concurrently, another trial (NCT06082713) focuses on the discovery of blood-based biomarkers for HD progression using extracellular vesicles. This study aims to enhance the precision of HD monitoring and establish standardized methodologies for extracellular vesicle biomarker research, thereby setting a foundational benchmark for the field. Together, these studies exemplify the dual focus of current exosome research on therapeutic application and biomarker development, highlighting the field’s growing impact on personalized medicine and its potential to revolutionize treatment paradigms for neurological disorders.

### 7.5. Future Directions

The unique ability of exosomes to cross the BBB offers a significant advantage for both diagnostics and therapeutics. This property has made exosomes a highly attractive tool for delivering therapeutic agents directly to the brain. Furthermore, their role in carrying disease-specific biomarkers presents a non-invasive approach to diagnosing multiple CNS disorders such as CVD, tumors, and neurodegenerative diseases. However, challenges remain in standardizing protocols for exosome isolation, achieving consistent targeting precision, and ensuring sufficient payload capacity. Current studies are also limited by technical bottlenecks in large-scale production and challenges in ensuring long-term safety and biocompatibility. Additionally, the complexity of exosome-mediated signaling in the CNS is not yet fully understood, limiting the translation of basic research findings into clinical applications.

To navigate these challenges, future research directions must prioritize the integration of cutting-edge technologies in nanotechnology and synthetic biology. These disciplines hold promise for the creation of engineered exosomes with enhanced stability, targeting precision, and therapeutic payload delivery. Fostering interdisciplinary collaborations and public-private partnerships will be crucial for bridging the gap between exosome research and its clinical implementation. Such collaborations can accelerate the translation of basic research findings into therapeutic innovations, paving the way for exosome-based diagnostics and therapeutics in CNS disease management. Additionally, standardizing protocols for exosome production, purification, and characterization across laboratories will be critical for ensuring reproducibility and scalability.

## 8. Conclusions

While exosomes present a novel and promising avenue for the diagnosis and treatment of CNS diseases, realizing their full potential requires addressing current technical, therapeutic, and regulatory challenges. Through concerted efforts in research and development, standardization, and collaboration, exosomes can emerge as a cornerstone of future therapeutic strategies against CNS pathologies.

## Figures and Tables

**Table 1:** The main effects of exosomes in the progression of cerebrovascular diseases.

Targeted Disease	Parental Cell	Effective Elements of Exosome	Target Cell	Effect	Possible Mechanisms	Ref.
Ischemic cerebrovascular disease	NPC/EPC	miR-126/miR-210	Nerve cell	Neuroprotection	Downregulation of Nox2 and increase in BDNF and p-TrkB/TrkB levels	[53]
Ischemic cerebrovascular disease	M2 microglia	OIP5-AS1	Nerve cell	Neuroprotection	Induce TXNIP ubiquitination and degradation by recruiting ITCH, negatively regulating TXNIP stability	[54]
Ischemic cerebrovascular disease	ADSC	miR-760-3p	Nerve cell	Neuroprotection	Inhibit the expression of CHAC1 to restrain ferroptosis	[55]
Ischemic cerebrovascular disease	Tumor cell	PD-L1	T cell	Suppress inflammation	Exosomal cargoes such as PD-L1 can retard necrosis, alleviating the burst of DAMPs at its source	[61]
Ischemic cerebrovascular disease	Microglia	MPO	Nerve cell	Trigger inflammation	Stimulate the production of hypochlorous acid and oxidative modification of HMGB1, facilitating the secretion and release of HMGB1 and activating neuroinflammation	[62]
Ischemic cerebrovascular disease	Panax notoginseng	Lipid	Nerve cell	Suppress inflammation	Alter the phenotype of microglia from “pro-inflammation” M1 type to “anti-inflammation” M2 type	[64]
Ischemic cerebrovascular disease	Neuron	miR-21-5p	Microglia	Trigger inflammation	Exosomal miR-21-5p induces microglia polarization, aggravating the release of neuroinflammation factors	[65]
Ischemic cerebrovascular disease	BMEC	miR-3613-3p	Microglia	Trigger inflammation	Exosomal miR-3613-3p promotes microglial M1 polarization by inhibiting RC3H1 protein levels	[66]
Ischemic cerebrovascular disease	/	CpG oligonucleotides	Macrophage	Trigger inflammation	Act on toll-like receptors to activate the NF-κB signaling pathway, thus promoting the polarization of macrophages to the M1 phenotype	[68]
Ischemic cerebrovascular disease	/	/	Macrophage	Suppress inflammation	Activate the PGE2/CREB pathway, thus decreasing the production of inflammatory cytokines	[70]
Ischemic cerebrovascular disease	Macrophage	Unspecified	VSMC	Angiogenesis	Stimulate JNK and p38 pathways to enhance the production of MMP-2 in VSMCs	[73]
Ischemic cerebrovascular disease	Endothelial cell	VEGF	Endothelial cell	Angiogenesis	Bind to the relevant receptor and then activate MAPK	[74]
	MSC	miR-21	Endothelial cell	Angiogenesis	Upregulate vascular growth factor levels via the PTEN/Akt pathway	[77]
Ischemic cerebrovascular disease	MSC	Unspecified	Pericyte	Angiogenesis	Inhibit pericyte pyroptosis	[79]
Ischemic cerebrovascular disease	Nerve cell	BDNF	Nerve cell	Neuroprotection	Improve the pathological microenvironment and promote neuroplasticity	[84]
Ischemic cerebrovascular disease	Nerve cell	miR-124-3p	Nerve cell	Neuroprotection	Upgrade the level of MYH9 by PI3K/AKT/NF-κB signaling cascades to suppress the activation of M1 microglia	[85]
Hemorrhagic cerebrovascular disease	MSC	miR-193b-3p	Nerve cell	Suppress inflammation	Reduce the expression of HDAC3 and acetylate the NF-κB p65	[91]
Hemorrhagic cerebrovascular disease	Neuron	miR-124	Microglia	Suppress inflammation	Target protein CCAAT-enhancer-binding protein α	[92]
Hemorrhagic cerebrovascular disease	MSC	BDNF	Nerve cell	Neuroprotection	Facilitate the expression of the anti-apoptotic protein gene BCL-2, thus promoting neuronal cell survival and increasing synaptic plasticity	[93]

Abbreviations: NPC, neural progenitor cell; EPC, endothelial progenitor cell; Nox2, NADPH oxidase 2; BDNF, brain-derived neurotrophic factor; TrkB, tyrosine receptor kinase B; OIP5-AS1, opa interacting protein 5-antisense RNA 1; TXNIP, thioredoxin interacting protein; ITCH, itchy E3 ubiquitin protein ligase gene; ADSC, adipose-derived stem cell; CHAC1, ChaC glutathione specific gamma-glutamylcyclotransferase 1; PD-L1, programmed cell death 1 ligand 1; DAMP, damage associated molecular pattern; MPO, myeloperoxidase; HMGB1, high mobility group box-1 protein; BMEC, brain-microvessel endothelial cell; RC3H1, ring finger and CCCH-type domains 1; NF-κB, nuclear factor kappa-B; PGE2, prostaglandin E2; CREB, cAMP-response element binding protein; VSMC, vascular smooth muscle cell; JNK, c-Jun N-terminal protein kinases; MMP-2, matrix metalloproteinase-2; VEGF, vascular endothelial growth factor; MAPK, mitogen-activated protein kinase; MSC, mesenchymal stem cell; PTEN, phosphatase and tensin homolog deleted on chromosome ten; Akt, protein kinase B; MYH9, myosin heavy chain 9; PI3K, phosphoinositide 3-kinase; HDAC3, histone deacetylase 3; BCL-2, B-cell lymphoma-2.

**Table 2:** The main effects of exosomes in the progression of brain tumors.

Parental Cell	Effective Elements of Exosomes	Target Cell	Effect	Possible Mechanisms	Ref.
Tumor cell	VEGF/FGF/IL-6/IL-8	Endothelial cell	Angiogenesis	Stimulate the migration and proliferation of endothelial cells	[14]
Tumor cell	miR-135b	Endothelial cell	Angiogenesis	Block the expression of HIF-1	[112]
Tumor cell	miR-210	Endothelial cell	Angiogenesis	Stimulate hypoxic signaling	[112]
Tumor cell	miR-21	Endothelial cell	Angiogenesis	Upregulate the expression of pro-angiogenic factors	[112]
Glioma cell	Notch1 Protein	Glial cell	Carcinogenesis	Deliver Notch1 to surrounding cells, inducing their dedifferentiation to glioblastoma stem cells	[117]
Mesenchymal stem cell	Unspecified	Treg cell	Suppress inflammation	Restrain the expression of inflammatory cytokines and modulate immune responses by increasing the production of anti-inflammatory mediators/regulate the Treg population	[119]
Tumor cell	PD-L1	CD8+ T cell	Immunoregulation	Exosomal PD-L1 binds with PD-1 on the CD8+ T cell surface, thus attenuating CD8+ T cell function	[122]
Tumor cell	Galectin-1	CD8+ T cell	Immunoregulation	Induce a SP in CD8+ T cells	[123]
Tumor cell	Functional receptor tyrosine kinase	Monocyte	Immunoregulation	Activate the MAPK pathway and generate a block of caspase cleavage	[125]
Tumor cell	circ_0072083	Tumor cell	Drug resistance	Regulate miR-1252-5p-mediated degradation and demethylation to enhance the expression of NANOG	[127]

Abbreviations: VEGF, vascular endothelial growth factor; FGF, fibroblast growth factor; HIF-1, hypoxia-inducible factor-1; Notch1, neurogenic locus notch homolog protein 1; PD-L1, programmed cell death 1 ligand 1; SP, suppressor phenotype; MAPK, mitogen-activated protein kinase; NANOG, Nanog homeobox.

**Table 3:** The main effects of exosomes in the progression of Alzheimer’s Disease.

Parental Cell	Effective Elements of Exosomes	Target Cell	Effect	Possible Mechanisms	Ref.
Neuron	miR-101	/	Propagate Aβ	Inhibit amyloid precursor protein expression and β-secretase activity	[143]
Neuron	Alix	/	Propagate Aβ	Unspecified	[144]
Neuron	miR-200c	Neuron	Increase Tau phosphorylation	Promote p-GSK-3β phosphorylation	[147]
Neuron	Metabolites of Aβ	Neuron	Neuronal loss	Damage cellular mitochondria as well as regulate ion channels, thus promoting the death of the cells involved	[152,153]
Neuron	LRP6/HSF1	Neuron	Neuroprotection	Activate genes encoding survival factors	[154]
Astrocyte	Complement protein	Macrophage/Neutrophil	Inflammation	Stimulate inflammatory cells	[155]

Abbreviations: Aβ, amyloid β-protein; p-GSK-3β, glycogen synthase kinase-3β phosphorylation; LRP6, low-density lipoprotein receptor-related protein 6; HSF1, heat-shock factor-1.

**Table 4:** The main effects of exosomes in the progression of Parkinson’s disease, Huntington’s disease, and multiple sclerosis.

Parental Cell	Effective Elements of Exosomes	Target Cell	Effect	Possible Mechanisms	Ref.
Microglia	a-synuclein	Neuron	Transmission of a-synuclein	Exosomes fuse with target cells	[172]
Microglia	miR-21/miR-146a	Neuron	Inflammation	Stimulate the NF-κB/NLRP3 pathway	[175]
Neuron	Huntington protein	Neuron	Transmission of Huntington protein	Exosomes fuse with target cells	[186]
Unspecified	Let-7i	Foxp3+Treg cells	Immunomodulation	Block the IGF1R/TGFBR1 pathway	[187]

Abbreviations: NF-κB, nuclear factor kappa-B; NLRP3, nucleotide-binding oligomerization domain-like receptor protein 3; IGF1R, insulin-like growth factor 1 receptor; TGFBR1, transforming growth factor beta receptor 1.

**Table 5:** Ongoing clinical research on exosomes.

Research Period	Related Disease	Possible Exosome Effects	Research Purpose	Study Type	Estimated Enrollment	NCT Number
2023/10/25–2031/11	HD	Biomarker	Use EVs to identify a less invasive blood-based biomarker of brain Huntingtin	Observational	100 participants	NCT06082713
2022/11/18–2026/01/31	Acute ischemic cerebrovascular syndrome	Biomarker	Determine the performance of EV profiling, added to a structured clinical and imaging evaluation, to discriminate TIAs from TIA mimics	Observational	200 participants	NCT06319742
2021/10/01–2027/09/01	Acute ischemic cerebrovascular syndrome	Biomarker	Determine whether the exosomes derived from endothelial cells increase thrombotic risk and the resulting extended prothrombin time is associated with patients at highest risk of stroke	Observational	360 participants	NCT05645081
2021/04/01–2025/06/01	Post-stroke cognitive impairment	Biomarker	Investigate capillary dysfunction and exosome profiles as predictors of cognitive function one year after AIS and TIA	Observational	140 participants	NCT06257823
2022/07/06–2023/12/31	PD	Biomarker	(1) Compare the effects between experimental treatment and conventional treatment; (2) Explore whether it is possible to identify predictive and indicative biomarkers of an outcome measure of rehabilitation using EVs	Interventional (Clinical Trial)	60 participants	NCT05902065
2017/12/20–2023/12	AD	Biomarker	Explore the presence of Tau in extracellular vesicles in CSF	Observational	100 participants	NCT03381482
2013/01–2016/06/21	PD	Biomarker	(1) Determine whether there are biomarkers associated with PD susceptibility and/or progression in exosome-proteomes; (2) Determine if LRRK2 expression and/or phosphorylation are significantly lowered in the exosomes of individuals treated with the potent LRRK2 kinase inhibitor sunitinib (a multi-kinase inhibitor compound), to establish an assay for on-target effects for future LRRK2 inhibitor clinical trials	Observational	601 participants	NCT01860118
2022/08/01–2025/07/31	PSD	Treatment	Explore the role of acupuncture-induced exosomes in the treatment of PSD	Interventional	30 participants	NCT05326724
2024/05/27–2025/08/30	AIS	Treatment	Evaluate safety and preliminary efficacy of intravenous exosomes derived from human induced pluripotent stem cells in AIS	Interventional	29 participants	NCT06138210
2023/07/04–2024/05/31	OMG	Biomarker	Find some specific miRNAs to diagnose OMG (miR-340-5p, miR-106b-5p, or miR-27a-3p)	Observational	160 participants	NCT05888558
2024/12–2034/12	AD	Prevention	Investigate the effect of a long-term combined aerobic exercise and cognitive training program on cognitive function and blood exosomal synaptic protein levels for seniors at increased risk for AD	Interventional	200 participants	NCT05163626

Abbreviations: HD, Huntington’s disease; EVs, extracellular vesicles; TIAs, transient ischemic attacks; AIS, acute ischemic stroke; PD, Parkinson’s Disease; AD, Alzheimer’s Disease; CSF, cerebrospinal fluid; LRRK2, leucine-rich repeat kinase 2; PSD, post-stroke depression; OMG, ocular myasthenia gravis.

## Data Availability

Not applicable.

## Abbreviations

CNS	Central nervous system
BBB	Blood-brain barrier
ESEs	Early sorting endosomes
LSEs	Late sorting endosomes
MVBs	Multivesicular bodies
ESCRT	Endosomal sorting complex required for transport
CVD	Ischemic cerebrovascular disease
Nox2	NADPH oxidase 2
ROS	Reactive oxygen species
BDNF	Brain-derived neurotrophic factor
TrkB	Tyrosine kinase receptor B
OIP5-AS1	Opa interacting protein 5-antisense RNA 1
TXNIP	Thioredoxin-interacting protein
CHAC1	ChaC glutathione specific gamma-glutamylcyclotransferase 1
DAMPs	Damage-associated molecular patterns
PD-L1	Programmed death-ligand 1
IRF4	Interferon regulatory factor 4
NF-κB	Nuclear factor-kappa B
TLR	Toll-like receptor
PGE2	Prostaglandin E2
CREB	cAMP response element-binding protein
MMPs	Matrix metalloproteinases
JNK	c-JunN-terminal kinase
EC	Endothelial cell
VEGF	Vascular endothelial growth factor
PTEN	Phosphatase and tensin homolog deleted on chromosome ten
Akt	Protein kinase B
PI3K	Phosphoinositide 3-kinase
ICH	Intracerebral hemorrhage
SAH	Subarachnoid hemorrhage
HDAC3	Histone deacetylase 3
MSC	Mesenchymal stem cell
CVT	Cerebral venous thrombosis
ALOX15	Arachidonate-15-Lipoxygenase
VTE	Venous thromboembolism
TME	Tumor microenvironment
FGF	Fibroblast Growth Factor
IL-6	Interleukin-6
IL-8	Interleukin-8
VEC	Vascular endothelial cell
TGF-β	Transforming growth factor beta
IL-4	Interleukin-4
IL-10	Interleukin-10
PD-1	Programmed death-1
AD	Alzheimer’s disease
PD	Parkinson’s disease
MS	Multiple sclerosis
HD	Huntington’s disease
Aβ	Amyloid-beta
NFT	Neurofibrillary tangle
LRP6	Low-density lipoprotein receptor-related protein 6
HSP-1	Heat shock protein-1
CSF	Cerebrospinal fluid
α-syn	α-synuclein
NLRP3	NLR family pyrin domain containing 3
IGF1R	Insulin-like growth factor 1 receptor
TGFBR1	TGF-β type 1 receptor
